# Sorption Properties of Specific Polymeric Microspheres towards Desethyl-Terbuthylazine and 2-Hydroxy-Terbuthylazine: Batch and Column Studies

**DOI:** 10.3390/ma14112734

**Published:** 2021-05-22

**Authors:** Sylwia Ronka, Weronika Bodylska

**Affiliations:** Faculty of Chemistry, Wrocław University of Science and Technology, Wybrzeże Wyspiańskiego 27, 50-370 Wrocław, Poland; 233588@student.pwr.edu.pl

**Keywords:** polymeric adsorbent, hydrogen bonding, adsorption, persistent and mobile chemicals, herbicides

## Abstract

This work investigates the sorption properties of poly(divinylbenzene) modified in the Diels–Alder reaction towards persistent and mobile metabolites of terbuthylazine. The batch experiments were carried out to examine the efficiency of desethyl-terbuthylazine and 2-hydroxy-terbuthylazine adsorption on the specific adsorbent and the impact of different factors on the adsorption process. Results fit well to a pseudo-second order kinetic model. It was confirmed that hydrogen bonds play an important role in the studied systems. Five times greater sorption of 2-hydroxy-terbuthylazine than desethyl-terbuthylazine was observed. The molecular structures of both metabolites exhibit complementarity to the arrangement of functional groups in the polymer but the differences in the physicochemical properties of the desethyl derivative make it a highly mobile compound with higher affinity to the aqueous phase. The equilibrium data in the batch study fit the Freundlich isotherm for 2-hydroxy-terbuthylazine, and for desethyl-terbuthylazine the Temkin and Dubinin–Radushkevich models were better. The adsorption capacities obtained under dynamic conditions were comparable with batch results. For column adsorption modeling the Bohart–Adams, Wolborska, Thomas and Yoon–Nelson models were used. The proposed microspheres can be reused easily with no significant decrease in adsorption capacity by using ethanol as eluent in the desorption.

## 1. Introduction

Herbicides, as well as their degradation products, are toxic substances that can easily remain for many years in various environmental compartments due to their high chemical and biochemical stability in the environment. Agricultural soils are the first recipients of herbicides after their application. When plant protection products bind strongly to soil, which is mainly the case with hydrophobic and cationic compounds, their persistence there increases, while biodegradability becomes lower. On the other hand, the low adsorption of herbicides in soil greatly facilitates their migration to surface waters and groundwaters [1,2,3]. Therefore, herbicides are capable of creating residues that can be detected in the environment even after a long time [1].

Contaminants that are poorly degradable and easily moved to a wider area by spreading through the environment (groundwater, surface waters, bottom sediments) are defined as persistent and mobile compounds [4,5,6]. According to the first pan-European reconnaissance of the occurrence of polar organic persistent pollutants in European groundwater [7], terbuthylazine and desethyl-terbuthylazine were among the pollutants that exceeded the European Union (EU) groundwater standard of 0.1 μg L^−1^ [8]. A study was conducted for 59 selected compounds, including pharmaceuticals, pesticides and their transformation products, perfluorinated acids (PFAs), benzotriazoles and others. In total, 164 samples from 23 countries were analyzed, detecting the desethyl-terbuthylazine in 49% of samples (maximum concentration was 266 ng L^−1^) and the terbuthylazine in 34% of samples (maximum concentration was 716 ng L^−1^).

Terbuthylazine became one of the most widely used herbicides in crops protection, mainly due to the ban on the use of atrazine in Europe from 2004. It is known that this substance presents a potential risk due to its combination of persistence and toxicity to living organisms. There is a risk, that long-term exposure of aquatic organisms, mammals and soil invertebrates to high concentrations of this pesticide may cause negative effects such as DNA damage and carcinogenic effects [9,10]. Moreover, some studies show that the toxicity of the herbicide metabolites may be higher than their parent compound [11,12]. TBA mineralization and transformation rely on two processes—chlorohydrolysis, with associated depletion of the chlorine atom or N-dealkylation of the side chains of the s-triazine ring. The main degradation pathways of TBA in the soil are presented in [13]. Depending on the microbial activity, metabolites such as desethyl-terbuthylazine, hydroxy-terbuthylazine, desethylhydroxy-terbuthylazine, diamino-chlorotriazine), desbutylhydroxy-terbuthylazine, amino-dihydroxy-triazine may be formed. Desethyl-terbuthylazine and 2-hydroxy-terbuthylazine are the major products of degradation of terbuthylazine in soil [14] and the desethyl-terbuthylazine is the most detected terbuthylazine metabolite in surface waters [15,16]. Few studies show the negative effects of desethyl-terbuthylazine and 2-hydroxy-terbuthylazine on aquatic organisms [17,18,19]. However, as classification with R40 (carcinogen category 3) is proposed for terbuthylazine, the groundwater metabolites are considered toxicologically relevant [20]. 

The pesticide transformation products often show higher persistence and mobility than the active substance [21]. The persistence in the soil for terbuthylazine is medium to high—the period required for 50% dissipation (DT50) is 65–167 days (20 °C, soil moisture 13–36% *w*/*w*). DT50 for desethyl-terbuthylazine is 27–113 days (20 °C, soil moisture 11–29% *w*/*w*) and for 2-hydroxy-terbuthylazine is 207 to >1000 days (20 °C, soil moisture 11–29% *w*/*w*), so they exhibit moderate to high persistence and high to very high persistence, respectively [22]. Regarding mobility, 2-hydroxy-terbuthylazine and terbuthylazine are classified as medium mobile, while desethyl-terbuthylazine is highly mobile [22]. Therefore, terbuthylazine metabolites as persistent and mobile chemicals are frequently detected in the environmental compartments in Europe, in many cases at concentrations higher than the European drinking water limit according to Directive (EU) 2020/2184 [23] equal to 0.1 µg L^−1^, e.g., in the range of up to 9.24 μg L^−1^ in surface waters for 2-hydroxy-terbuthylazine and 2.90 μg L^−1^ and 3.50 μg L^−1^ for desethyl-terbuthylazine in surface water and groundwater, respectively [24,25,26,27]. Desethyl-terbuthylazine was also often found in tap water, for example in samples collected in France [28] and Croatia [29]. Regarding the above, Tasca and Fletcher signal that the range of emerging contaminants detected in drinking waters, and their seasonal and geographic variability, demands more accurate screening programs, and greater attention has to be focused on the fate of herbicide metabolites [30]. This is especially important in the case of toxic substances. However, it must be noted that also contamination with less toxic but persistent and mobile substances can become biologically relevant. Once released into the environment, these chemicals may spread easily to a wider area of the environment or adsorb on the bottom sediments of reservoirs or soil particles. On the one hand, adsorption is beneficial because it reduces the bioavailability of these compounds so that they are not readily absorbed by the organisms of a given ecosystem. On the other hand, adsorbed substances are accumulated and gradually released into water bodies. Therefore, developing the aquatic system and wastewater monitoring as the main source of persistent and mobile substances is one of the European Union’s objectives, which allows the risk for human health to be limited and the life cycle assessment of these substances in the environment to be determined. 

However, a substantial analytical challenge exists related to the detection and quantification of persistent and mobile chemicals in the environmental samples. It is very important, especially for those substances that have not previously been covered in the routine monitoring, which also include desethyl-terbuthylazine and 2-hydroxy-terbuthylazine. As long as persistence and toxicity are not of great importance regarding chemical analysis, the mobility significantly affects analytical processes applied in the trace analysis of organic contaminants that are present in the water environment [31]. 

The analysis of trace impurities is preceded by the stage of preconcentration of targeted compound, most often with the use of the solid-phase extraction (SPE) method, using various types of adsorbent. It should also be emphasized that the persistent and mobile organic compounds are more of a concern for water as they are difficult to capture by sorption processes due to their high polarity [5]. Herbicide metabolites detection is also difficult due to their low concentrations in the environmental compartments (ng L^−1^) and much higher concentrations (mg L^−1^) of other pollutants occurring in environmental samples. In order to overcome all these difficulties, the specific sorptive materials for SPE should be used, the effectiveness of which will be increased by selective interactions with the functional groups of individual types/groups of persistent and mobile chemicals. Terbuthylazine metabolites belong to the group of triazines having both hydrogen acceptor and hydrogen donor, indicating a high potential for the formation of hydrogen bonds which, besides van der Waals interactions, play important roles in the sorption of this group of herbicides. 

Nowadays, the most common method for the removal of contaminants from aquatic environments is adsorption on polymeric materials [32,33,34]. Our previous study revealed that poly(divinylbenzene) modified in the Diels–Alder reaction with maleic anhydride and subsequent hydrolysis can be successfully used for removal of terbuthylazine from water solutions [35,36], also from multi-component systems [37]. Carboxyl groups, implemented into the adsorbent surface, create centers of specific interactions such as hydrogen bonding between the adsorbate and the adsorbent, which resulted in intensification and selectivity of adsorption. The next study showed that the proposed microspheres was equally effective in real solutions, containing significant amounts of other substances typical for agricultural wastewater, which is important from the point of view of its practical application, for example, in systems of agricultural wastewater treatment [38].

Since desethyl-terbuthylazine and 2-hydroxy-terbuthylazine are derivatives of terbuthylazine and there is a visible structural similarity, using poly(divinylbenzene) modified with maleic anhydride for adsorption of these compounds therefore seems to be a suitable option. In this work, the batch and column experiments were carried out to examine the efficiency of desethyl-terbuthylazine and 2-hydroxy-terbuthylazine adsorption on specific polymeric microspheres and the impact of different factors such as pH and temperature on this process. Moreover, the aim of this study is to examine the kinetics and mechanism of terbuthylazine metabolites adsorption in the proposed adsorbate–adsorbent systems. 

## 2. Materials and Methods

### 2.1. Materials

Analyzed herbicides: desethyl-terbuthylazine and 2-hydroxy-terbuthylazine (purities ≥ 99%) were obtained from Dr. Ehrenstorfer GmbH (Augsburg, Germany). Millipore deionized water was used for making the solutions during sorption experiments. Ethanol (96%) and sodium hydroxide were purchased from P.P.H. “STANLAB” Sp. J. (Lublin, Poland).

Polymeric microspheres were made in our laboratory by modification of the pendant vinyl groups of poly(divinylbenzene) by maleic acid anhydride through the Diels–Alder reaction. Polymer synthesis and modification were carried out according to the previously published procedures [39,40].

### 2.2. Sorption Studies 

The physicochemical properties of analyzed terbuthylazine metabolites are presented in Table 1.

#### 2.2.1. Batch Studies

Different amounts of wet-weighed polymer adsorbent were contacted with 20 mL of 10 mg L^−1^ ethanol/water solutions (1/9, *v*/*v*) of terbuthylazine metabolites at pH 3, 5 and 7 by continuous shaking at 20, 35 and 50 °C for 48 h. The concentrations of terbuthylazine metabolites were measured using ultraviolet–visible (UV/VIS) spectroscopy, Jasco V-630 apparatus (JASCO EUROPE s.r.l., Cremella, Italy) The wavelength was set at 215.0 for desethyl-terbuthylazine and 238.0 nm for 2-hydroxy-terbuthylazine. The value of terbuthylazine metabolite sorption capacity (*q_eq_*), expressed in milligrams of adsorbate per gram of dry adsorbent, was calculated from the mass balance and the initial concentration of metabolite in the solution. The distribution coefficients (*K*) were calculated as the ratio of the amount of metabolite adsorbed by 1 g of a dry polymer and the amount of metabolite remaining in 1 mL of solution at equilibrium after sorption. Sorption kinetics was examined at 20 °C by mixing adsorbent with herbicide metabolite solutions (C = 10 mg L^−1^, pH = 7), collecting samples at fixed times and analyzing the residual concentration of metabolite. 

#### 2.2.2. Fixed Bed Sorption Studies

Dynamic sorption was carried out in fixed bed columns (diameter 0.9 cm) filled with 0.3 g of tested sorbent. Sorption was carried out by passing downwards a terbuthylazine metabolites solutions (C = 10 mg L^−1^, pH = 7) with a flow rate 0.9 mL min^−1^. Samples were collected at regular time intervals (15 min) using a fraction collector and the outlet concentration was measured by UV/VIS spectroscopy. For desorption, a similar procedure was used using 96% ethanol. The sampling time was decreased to 5 min and the process was carried out until full elution of the metabolites. Five consecutive sorption/desorption cycles for desethyl-terbuthylazine and three sorption/desorption cycles for 2-hydroxy-terbuthylazine were used for determining the reusability of microspheres. All of the experiments were carried out at room temperature (20 °C). 

In order to analyze experimental data, the terbuthylazine metabolites’ adsorption breakthrough curves were determined by plotting the effluent metabolite concentration (C) versus the effluent volume (V). The calculations of total and usable adsorptive capacity, mass transfer zone and mass exchange zone moving rate were made based on the methodology described in detail in reference [46]. 

## 3. Results and Discussion

### 3.1. Adsorbent Characterization

**Figure 1:**
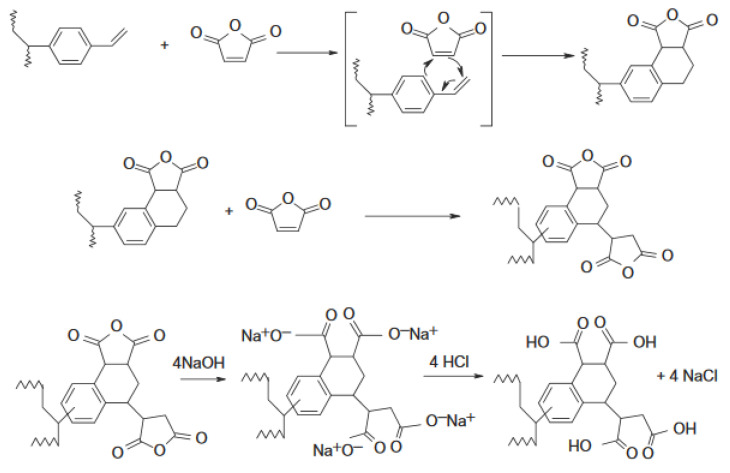
Scheme of poly(divinylbenzene) modification using maleic anhydride [47].

The proposed microspheres for terbuthylazine metabolites sorption were designed in such a way that they have sets of donor and acceptor atoms complementary to the structures of triazines molecules. For this purpose, synthesized by radical suspension polymerization poly(divinylbenzene) microspheres were modified in the Diels–Alder reaction (see Figure 1). Carboxyl groups, which are capable of creating specific interactions, were obtained after opening the ring of maleic anhydride with the use of base hydrolysis and cyclization with hydrochloric acid. 

**Figure 2:**
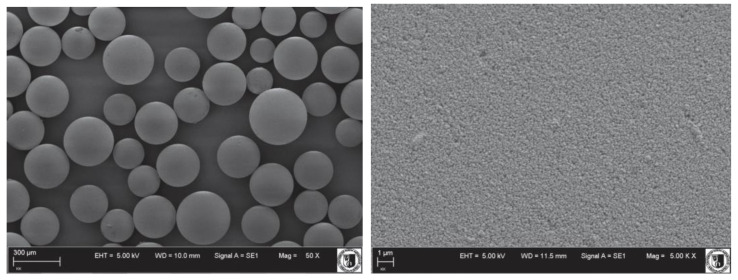
Scanning electron microscope (SEM) images of modified poly(divinylbenzene) beads.

Selected adsorbent has a predominantly mesoporous structure with an average pore size 6.62 nm and a total pore volume 0.96. Its scanning electron microscope (SEM) micrographs (ZEISS, Zeiss EVO LS 15, Oberkochen, Germany) are shown in Figure 2. The studied polymeric microspheres are characterized by a well-developed surface area (578 m^2^ g^−1^) and good water regain (2.54 g g^−1^), which have a direct impact on the adsorption efficiency. 

The carboxylic group content in the structure of the proposed adsorbent results from the effectiveness of modifying free vinyl groups of poly(divinylbenzene). Whereas the number of vinyl groups that can be modified depends on the polymerization conditions. Many attempts have been made to synthesize poly(divinylbenzene) microspheres in order to obtain as many sites as possible for reacting with maleic anhydride. Samples characterized by a lower amount of pendant vinyl groups, that could be modified, have a higher degree of crosslinking and at the same time lower carboxylic groups content. On the other hand, the degree of crosslinking is responsible for the appropriate structure and strength of the polymer microspheres. The SEM micrographs of the tested adsorbent (Figure 2) and the characteristics of its porous structure indicate that the selection of polymerization conditions was correct. The content of carboxyl groups in the tested polymer is 3.58 mmol g^−1^. It should be emphasized that the commercially available adsorbents have usually about 1 mmol g^−1^ of carboxylic groups (e.g., 0.74 mmol g^−1^ in Strata-X-CW 33u Polymeric Weak Cation from Phenomenex (Torrance, CA, USA) and so the content of carboxyl groups in the proposed microspheres is highly satisfactory. These groups can enlarge the capacity of sorption by the formation of specific interactions such as hydrogen bonds between the adsorbent and adsorbate. These interactions can be created between the hydroxyl hydrogen atom of the carboxylic group from modified polymer and the nitrogen atom from triazine, which contains free electron pair. Such interactions can be created also between the hydrogen atom of the triazine amino group and the carbonyl oxygen atom from the microspheres. The ability to the formation of these direct interactions indicates better adsorption and selectivity than that of traditional adsorbents. 

### 3.2. Batch Sorption Experiments

Sorption experiments were conducted for two metabolites of terbuthylazine: desethyl-terbuthylazine and 2-hydroxy-terbuthylazine. Modified with maleic anhydride, poly(divinylbenzene) was selected for sorption of these triazines due to structural similarity of derivatives to terbuthylazine, the sorption of which on this material was very effective [35]. Molecular structures of examined herbicides show complementarity to the configuration of functional groups of modified microspheres. Removal of triazines may arise from the formation of not only non-specific interactions but also from creating specific ones.

#### 3.2.1. Adsorption Kinetics 

**Figure 3:**
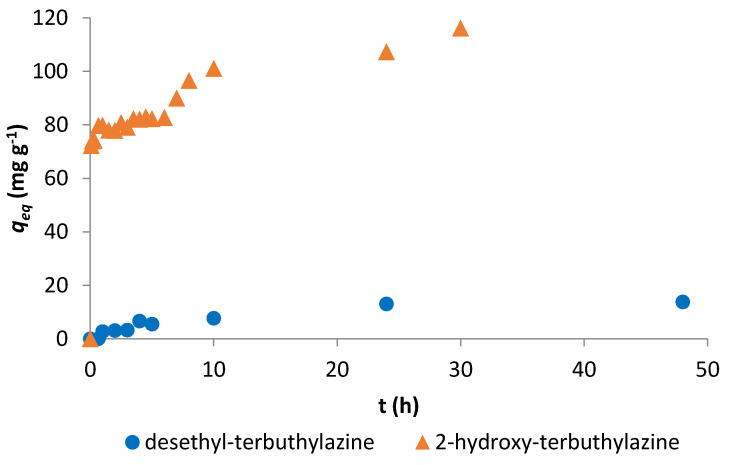
Sorption kinetics for desethyl-terbuthylazine and 2-hydroxy-terbuthylazine on modified poly(divinylbenzene) microspheres.

The time needed to reach the equilibrium in the metabolite adsorption process was determined in the batch adsorption experiment. The plots of the equilibrium sorption (*q_eq_*) versus time (*t*) are presented in Figure 3. It can be seen that sorption kinetics for the tested compounds are very different. The most intense adsorption shows 2-hydroxy-terbuthylazine for which the process efficiency is reached 56% after 5 min. For desethyl-terbuthylazine, a much slower process is observed. The sorption up to 56% is achieved after 10 h. Sorption equilibrium for both metabolites is reached after about 30 h. These kinetic results also confirm a much better affinity of the tested adsorbent for the hydroxyl metabolite than for terbuthylazine, the sorption kinetics of which was presented earlier [37]. 

The description of adsorption kinetics was carried out using two models: pseudo-first and pseudo-second order [48]. Equation (1) was used to determine the parameters of the pseudo-first order model:

(1) $$log\left(q_{eq}-q_{t}\right)=log(q_{eq})-k_{1}t/2.303$$

where *t* is time (min), $q_{eq}$ is sorption capacity at equilibrium (mg g^−1^),$\;q_{t}$ is sorption capacity in time (mg g^−1^), $k_{1}$ is pseudo-first order adsorption rate constant (min^−1^). The parameters of the pseudo-second order model were calculated using Equation (2):

(2) $$\frac{t}{q_{t}}=\frac{1}{k_{2}\cdot q_{eq}^{2}}+t/q_{eq}$$

where *t* is time (min), $q_{eq}$ is sorption capacity in equilibrium (mg g^−1^),$\;q_{t}$ is sorption capacity in time (mg g^−1^), $k_{2}$ is pseudo-second order adsorption rate constant (g mg^−1^ min^−1^). The results are presented in Table 2. 

Experimental data better correspond to the pseudo-second order model of kinetics. It confirms that adsorption of herbicides is a result of the specific interactions between triazines and surface of the adsorbent. The value of *q_eq_* is similar to the one obtained during sorption experiments (see Table 3). The calculated correlation coefficients R^2^ for this model are higher. In the case of the pseudo-first order model, *q_eq_* results do not reflect experimental values. The pseudo-first order equation is greatly influenced by random errors resulting from the experiment. In the case of a pseudo-second order equation, the impact of these errors is inconsiderable.

#### 3.2.2. Adsorption Isotherms

**Figure 4:**
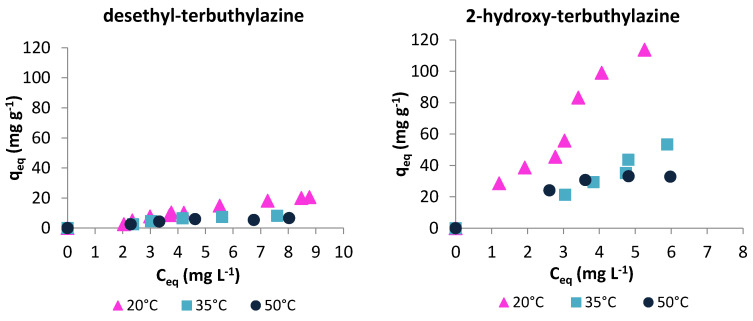
Adsorption isotherms for desethyl-terbuthylazine and 2-hydroxy-terbuthylazine at different temperatures: 20 °C, 35 °C and 50 °C.

Based on the batch studies the dependency of metabolites sorption on equilibrium concentration was plotted and shown in Figure 4. Analysis of the isotherms enabled the determination of the maximum sorption (*q_eq_*) and retention rate (*R*) for both terbuthylazine metabolites. It also allows determining the distribution coefficients (*K*) of triazines between the adsorbent and the adsorbate solution. All data were calculated based on results obtained for 10 mg L^−1^ metabolites solutions at 20 °C. Results are shown in Table 3.

The best sorption is shown by 2-hydroxy-terbuthylazine (113 mg g^−1^). It is much higher than the sorption of terbuthylazine (64 mg g^−1^) [37], which is the parent compound. Adsorption of organic substances may depend on the physicochemical properties of the adsorbate such as molecular weight, size and shape of a molecule, kind of functional groups, and solubility. For the compounds in question, these properties are similar, however the presence of OH group in the structure of 2-hydroxy-terbuthylazine may generate additional hydrogen bonds and therefore increase sorption efficiency. Referring to desethyl-terbuthylazine sorption, it was observed [35] that with the reduction of the presence of methyl groups in the structure of triazine-based herbicide, the capacity of sorption decreases, and due to that sorption of terbuthylazine is higher than desethyl-terbuthylazine (21 mg g^−1^). Five times greater sorption of 2-hydroxy-terbuthylazine than desethyl-terbuthylazine may also be the result of a greater contribution of non-specific sorption resulting from much lower solubility of 2-hydroxy-terbuthylazine (7.19 mg L^−1^) in water than desethyl -terbuthylazine (327.1 mg L^−1^). The calculated retention rates for both metabolites are similar, but the distribution coefficient for 2-hydroxy-terbuthylazine is significantly higher. 

Figure 4 also presents the sorption isotherms obtained at different temperatures (20, 35 and 50 °C). Adsorption is an exothermic process, which makes it less efficient as the temperature increases. Additionally, adsorption is affected not only by adsorbate-adsorbent interactions but also by interactions between the dissolved substance and the solvent, which are significantly influenced by temperature. As temperature increases, the solubility of the adsorbate increases, thus reducing its non-specific interactions with the adsorbent. It was observed for both terbuthylazine metabolites that with the increasing temperature of the process, the sorption efficiency decreased. This may also have been caused by the weakening of hydrogen bonds formed between groups in the structure of adsorbate and groups on the polymer surface. It can be seen that for 2-hydroxy-terbuthylazine the difference in adsorption at various temperatures is much bigger. It is likely that in the case of a metabolite having an additional group capable of forming hydrogen bonds (–OH), the specific interactions have a much greater influence on the effectiveness of adsorption process than non-specific interactions between adsorbent and adsorbate.

**Figure 5:**
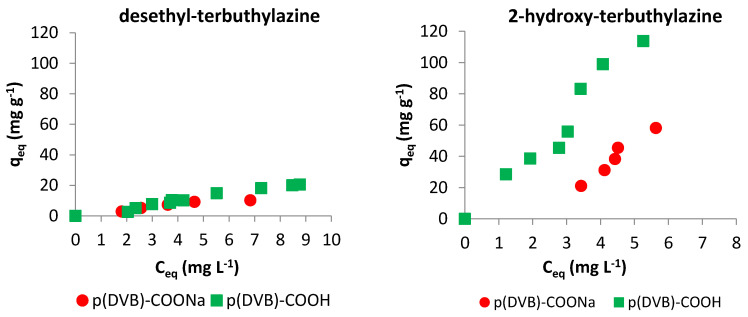
Adsorption isotherms for desethyl-terbuthylazine and 2-hydroxy-terbuthylazine on microspheres having carboxyl groups in acidic (p(DVB)-COOH) and sodium (p(DVB)-COONa) forms.

The role of hydrogen bonds in the sorption of terbuthylazine metabolites was also confirmed by a study in which the hydrogens of the adsorbent’s carboxyl groups were eliminated. For this purpose, 0.1 M sodium hydroxide was used, and after ion-exchange the polymer was washed using distilled water to neutral pH. The results of metabolites sorption on the microspheres having functional groups in the form of sodium salt and for comparison in the acidic form are presented in Figure 5. 

It can be seen that reducing the possibility of hydrogen bonding between the hydroxyl hydrogen atom of the carboxyl group from modified polymer and the nitrogen atom containing the free electron pair from adsorbate lowers the sorption efficiency by about 50%. The same effect was observed for the sorption of terbuthylazine [37].

**Figure 6:**
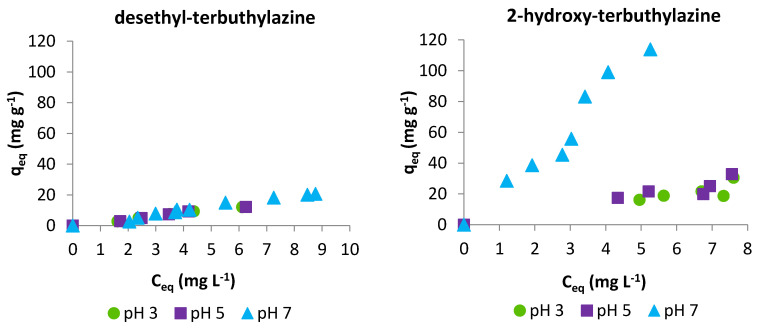
Adsorption isotherms for desethyl-terbuthylazine and 2-hydroxy-terbuthylazineat different pH: 3, 5 and 7.

The pH of the adsorbate solution has a huge effect on the adsorption process due to the possibility of affecting the interactions between adsorbate and adsorbent. Figure 6 presents adsorption isotherms for terbuthylazine metabolites obtained at different pH. 

In the case of desethyl-terbuthylazine, we do not observe any significant influence of pH on the sorption efficiency. Chlorinated triazines like desethyl-terbuthylazine are mainly weak bases and their logarithmic acid dissociation constant pK_a_ is approximately around 2 [43], and therefore in the range of studied pH (3–7) desethyl-terbuthylazine occurs in the form of inert molecules. The carboxylic groups on the surface of the adsorbent are mostly not dissociated (the pK_a_ value of the investigated polymer is around 6), so the possibilities for the formation of hydrogen interactions remain unchanged. In contrast, in the acidic pH range, 2-hydroxy-terbuthylazine displayed a markedly different behaviour than desethyl metabolite. The decrease in sorption efficiency at pH 5 and 3 is attributed to the high basicity of hydroxy triazines. With pK_a_ values around 5 [43], these compounds are protonated at lower pH, resulting in cationic species that can interact more easily with water molecules, increasing the hydration shell and at the same time weakening the interaction with the adsorbent. 

#### 3.2.3. Sorption Models

Four models such as Freundlich, Langmuir, Temkin and Dubinin–Radushkevich were used to match experimental data. Table 4 presents parameters of adsorption modeling using these calculations. The Langmuir model assumes monolayer adsorption on the polymer surface, which contains many active sites with equal energies that can only adsorb one adsorbate molecule each. This adsorption is often called ideal because the surface-active centers in the polymer are evenly distributed. This model is expressed as Equation (3):

(3) $$q_{eq}=q_{m}\cdot K_{\alpha }\cdot C_{eq}/\left(1+K_{\alpha }\cdot C_{eq}\right)$$

where: $q_{eq}$ is the amount of solute adsorbed per mass of adsorbent, $C_{eq}$ is the equilibrium concentration of solute in the solution. $K_{\alpha }$ i $q_{m}$ are characteristic of Langmuir equation (shown in Table 4) and were calculated from linearized form of Equation (3). The high R^2^ coefficient for desethyl-terbuthylazine makes it possible to conclude that the Langmuir model is well suited to describe the adsorption process of this triazine. For 2-hydroxy-terbuthylazine this factor is slightly lower. However, the determined $q_{eq}$ values are far too high compared to the experimentally obtained values.

The Freundlich model is used to describe non-ideal, reversible adsorption and is represented by the Equation (4):

(4) $$q=K_{F}\cdot C_{eq}^{1/n}$$

in which: *q* is sorption capacity at monolayer saturation (mg g^−1^), $C_{eq}$ is the equilibrium concentration of metabolite in aqueous solution, (mg L^−1^) $K_{F}$ is Freundlich’s constant and 1/*n* is the function of strength of the adsorption. $K_{F}$ determines the sorption capacity of the system and exponent 1/*n* indicates the variation of free enthalpy associated with adsorption from the solution. When 1/*n* is 1, the division between phases occurs independently of the herbicide concentration and the free enthalpy of the process is constant. A value of 1/*n* less than 1 suggests adsorption, in which the active centers have less and less free enthalpy. 1/*n* greater than 1 suggests cooperative adsorption, which assumes that increasing the number of adsorbate molecules on the surface of the adsorbent results in increased free enthalpy [49]. Characteristics were calculated using linearized form of Equation (4) and are presented in Table 4. The Freundlich model may also be suitable for describing the process of removing triazines from aqueous solutions due to the high correlation coefficients. For both studied metabolites, the 1/*n* factor is greater than 1, which suggests that cooperative sorption may occur in the system, in which the adsorbate absorbed earlier affects the adsorption of the remaining particles in the solution [49]. This phenomenon is very common in adsorption processes. 

The Temkin isotherm model includes interactions between adsorbent and adsorbate. It assumes that there is a linear decrease in the heat of adsorption of molecules in the layer, which is a result of an increase in the surface coverage. This isotherm model usually corresponds to a continuous, infinite (unlimited by minimum or maximum) energy distribution of adsorption sites. It also assumes that the process of adsorption is characterized by a uniform distribution of binding energies, up to maximum value [50]. The linear form of this model can be expressed as Equation (5):

(5) $$q_{e}=B_{T}ln\left(A_{T}\right)+B_{T}ln\left(C_{e}\right)$$

where: $B_{T}=RT/b_{T}$ is a constant related to the heat of adsorption, and $b_{T}$ (J mol^−1^) is Temkin isotherm constant, $q_{e}$ (mg g^−1^) is an amount of adsorbate in adsorbent at equilibrium, $A_{T}$ (L g^−1^) is Temkin isotherm equilibrium binding constant, T is the temperature (293 K) and R is the universal gas constant (8.314 J mol^−1^ K^−1^). The calculated results are presented in Table 4. Correlation coefficient for desethyl-terbuthylazine is the highest in this adsorption model, which may mean that this model describes best experimental data. The correlation coefficient for 2-hydroxy-terbuthylazine is definitely lower, which may mean that this model is not suitable for this system. For both studied metabolites, the value of the $b_{T}$ is positive, which confirms exothermic character of adsorption process.

**Figure 7:**
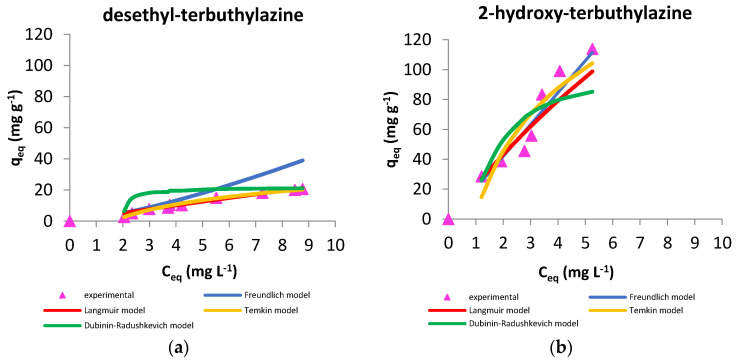
Experimental isotherms with predicted batch sorption model curves for desethyl-terbuthylazine (**a**) and 2-hydroxy-terbuthylazine (**b**).

The Dubinin–Radushkevich isotherm model is mainly applied to the mechanism of adsorption with Gaussian energy distribution onto heterogeneous surfaces. In this model, adsorption follows a pore filling mechanism. It assumes a multilayer character of the surface and involves van der Waals forces. It is also based on the value of the energy and provides information about the adsorption, whether it is chemical or physical [51]. When the parameter of mean free energy *E* is below 8 kJ mol^−1^ the adsorption process is considered as physical, whereas when parameter E is between 8 kJ mol^−1^ and 16 kJ mol^−1^ the process of adsorption is chemical [52]. Dubinin–Radushkevich isotherms were calculated using Equations (6)–(8):

(6) $$lnq_{e}=lnq_{s}-\left(K_{ad}\varepsilon^{2}\right)$$

(7) $$\varepsilon =RTln\left(1+\frac{1}{C_{e}}\right)$$

(8) $$E=1/\sqrt{2K_{ad}}$$

where: $q_{e}$ (mg g^−1^) is an amount of adsorbate in adsorbent at equilibrium, $q_{s}$ (mg g^−1^) is theoretical isotherm saturation capacity,$\;C_{e}$ (mg L^−1^) is adsorbate concentration at equilibrium, *E* (J mol^−1^) is mean free energy per molecule of adsorbate, $K_{ad}$ (mol^2^ J^−2^) and ε are Dubinin–Radushkevich constants. The calculated results are presented in Table 4. Values of mean free energy obtained for metabolites are lower than 8 kJ mol^−1^, which suggests that the process of adsorption is physical in both systems. Value of $q_{e}$ corresponds very well with experimental data for desethyl-terbuthylazine and correlation coefficient is high. For the other metabolite, 2-hydroxy-terbuthylazine, correlation coefficient is lower, however, the value of the $q_{e}$ is similar to the value obtained experimentally. Figure 7 shows experimentally obtained isotherms (pH = 7, T = 20 °C) for both metabolites with predicted model curves for every theoretical model.

The SSE parameter is known as the sum of the square estimate of errors and was obtained using software Origin Pro [53]. The obtained results are included in Table 4. The lowest values were obtained for Freundlich and Langmuir models for both studied systems. However, when deciding which model is the most suitable, the correlation coefficient should also be taken into consideration.

### 3.3. Fixed-Bed Column Studies

#### 3.3.1. Sorption Studies 

**Figure 8:**
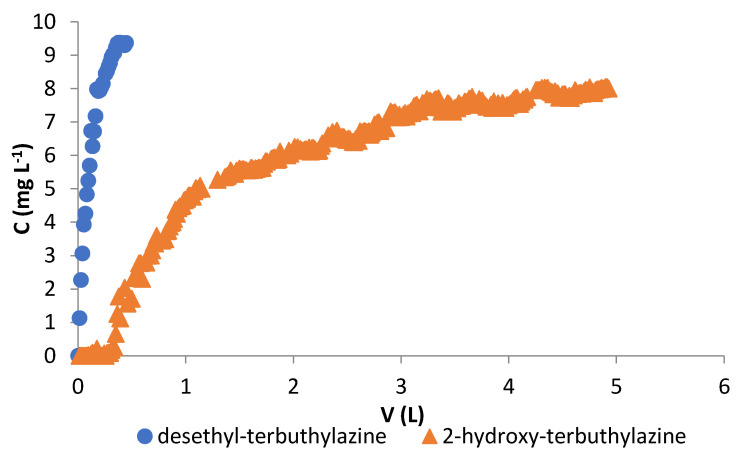
Adsorption breakthrough curves obtained in studied systems.

Sorption experiments were carried out in dynamic mode for both metabolites: desethyl-terbuthylazine and 2-hydroxy-terbuthylazine. The characteristics of the fixed bed are described by the breakthrough curve, which is the plot of adsorbate concentration in the effluent (C) versus effluent volume (V). Breakthrough curves plotted from the results obtained are presented in Figure 8. 

The analysis of the breakthrough curves clearly indicates that the tested specific adsorbent prefers sorption of 2-hydroxy-terbuthylazine, for which an extended breakthrough curve can be observed, indicating that a higher volume of metabolite solution could be treated. For desethyl-terbuthylazine the polymeric bed is rapidly saturated. Calculating the parameters of sorption on the basis of the breakthrough curve is an important issue because it provides the basic information for the design of the column adsorption system and can be used to determine the rational scale of the column adsorption for practical application. Breakthrough curves obtained for the tested metabolites can be described by mathematical equations presented in Table 5. Correlation coefficients are high and above 0.99 for both terbuthylazine derivatives. Characteristic parameters for dynamic sorption were calculated following the methodology described in the publication [46] and presented in Table 6.

Analysis of obtained data allows to determine not only the total (*P_t_*) and useful (*P_u_*) adsorption capacity, but also bed operation time until breakthrough (*t_b_*) and exhaustion (*t_e_*). The capacities of adsorption obtained under column studies are comparable to those obtained in batch adsorption systems (see Table 3). There is visible difference in time necessary for breakthrough and exhaustion of the column bed for used herbicide metabolites. The longer the time needed to reach those points the better filling of the polymer bed is observed. Time needed until breakthrough and until exhaustion are longer for 2-hydroxy-terbuthylazine, which may mean that the used microspheres have higher affinity for this metabolite. In fixed-bed experiments, the adsorbate concentration in the mobile phase as well as in the solid phase varies with time and position in the polymer bed. The mass transfer zone in the column moves from the top and towards to the bottom of the bed. In effective adsorption systems, low heights (*H*_0_) of mass transfer fronts and low mass exchange moving rate (*u*) are observed. Small values of this parameters mean that the breakthrough curve is close to an ideal step with negligible mass-transfer resistance [46]. 

The best adsorption parameters in fixed bed column studies were obtained for 2-hydroxy-terbuthylazine, due to the lower mass exchange moving rate and smaller height of adsorption front. The highest mass exchange moving rate for desethyl-terbuthylazine results probably from the weak affinity of this metabolite to the investigated polymer mainly because of its high water affinity (high water solubility—see Table 1). Therefore, also, the breakthrough and exhaustion times have the smallest values and consequently, the saturation of the adsorbent occurs earlier.

#### 3.3.2. Sorption Models

There are many fixed bed models, which can be used to describe the results obtained such as the Bohart–Adams model, Thomas model, Yoon–Nelson model or Wolborska model. Table 7 presents parameters of column adsorption modeling using these calculations. 

The Bohart–Adams model can be expressed by Equation (9):

(9) $$ln\left(\frac{C_{t}}{C_{0}}\right)=k_{AB}\cdot C_{0}\cdot t-k_{AB}\cdot N_{0}\cdot \left(\frac{Z}{U_{0}}\right)$$

where: $C_{0}$ and $C_{t}$ (mg L^−1^) are the initial and breakthrough concentration, $k_{AB}$ (L mg^−1^ min^−1^) is the Bohart–Adams kinetic constant, $N_{0}$ (mg L^−1^) is the saturation concentration, *Z* (cm) is the bed depth of the column, $U_{0}$ is the ratio of the volumetric flow rate (cm^3^ min^−1^) to the cross-sectional area of the bed (cm^2^). This model describes the relationship *C_t_*/*C*_0_ in a continuous system. It is usually used to express the initial part of the breakthrough curve. This model is assumed to follow a “step isotherm” in which the adsorption rate is proportional to the fractional adsorption capacity possible on the surface of the adsorbent. This concept is also consistent with the basic principle of surface reaction theory [54]. Values of $k_{AB}$ and $N_{0}$ were calculated from linearized plot of $ln\left(\frac{C_{t}}{C_{0}}\right)$ versus time (*t*). The value of the determined constant $k_{AB}$ is higher for desethyl-terbuthylazine, while the value of $N_{0}$ is higher for 2-hydroxy-terbuthylazine. Correlation coefficients *R*^2^ for both metabolites of terbuthylazine are low, which may mean that this model is not suitable for modeling of obtained data. It suggests that adsorption mechanism is complex and involves few rate limiting steps [55]. The Bohart–Adams model may be too simple to explain this adsorption mechanism. 

The Thomas model describes the adsorption process indicating that external and internal diffusion is not the limiting step. Thomas model is consistent with Langmuir adsorption and desorption kinetics. It is commonly used to calculate column performance and to predict breakthrough curves. It assumes negligible axial dispersion. The model is based on second order kinetics, which is the main limitation of this model. It does not constrain sorption by chemical reaction and is controlled by mass transfer at the surface. The Thomas model can be represented by Equation (10):

(10) $$ln\left(\frac{C_{0}}{C_{t}}-1\right)=k_{Th}\cdot q_{0}\cdot m/Q-k_{Th}\cdot C_{0}\cdot t$$

where: $C_{0}$ and $C_{t}$ (mg L^−1^) are the initial and breakthrough concentration, $k_{Th}$ (mL min^−1^ mg^−1^) is Thomas constant, $q_{0}$ (mg g^−1^) is the adsorption capacity, *t* (min) is the total flow time, *m* (g) is the amount of adsorbent placed in the column, *Q* (cm^3^ min^−1^) is the flow rate. Values of $k_{Th}$ and $q_{0}$ were calculated from linearized plot of $ln\left(\frac{C_{0}}{C_{t}}-1\right)$ versus time (*t*). Parameter $q_{0}$ does not correspond well with experimental data for both metabolites. The value of the Thomas kinetic constant $k_{Th}$ is greater for desethyl-terbuthylazine. Correlation coefficient for desethyl-terbuthylazine is high, however considering parameter $q_{0}$ this model is also not sufficient for obtained data. For 2-hydroxy-terbuthylazine correlation coefficient is definitely lower, which may mean this model is also not suitable. 

The Yoon–Nelson model is applied to a range of effluent concentrations between the column breakthrough and saturation times. It assumes that the rate of decrease in the adsorption probability of each adsorbate molecule is proportional to the adsorption capacity of the adsorbate molecules and the breakthrough probability of the adsorbent column [56]. This model is considered simple, thus it does not require any detailed data about the adsorbent such as type of material or properties [57]. Using this model, it is possible to minimalize error from the use of the Thomas model, which may occur at lower or higher periods of time of the breakthrough. This model is most suitable for sorption from single component solutions [54,58]. The Yoon–Nelson model may be expressed by Equation (11):

(11) $$ln\left(\frac{C_{t}}{C_{0}}-C_{t}\right)=k_{Yn}\cdot t-k_{Yn}\cdot \tau$$

where:$\;k_{Yn}$ (min^−1^) is the rate constant, τ (min) is the time needed for 50% adsorbate breakthrough. The linear plot of $ln\left(\frac{C_{t}}{C_{0}}-C_{t}\right)$ versus time (*t*) enables us to determine values of $k_{Yn}$ and τ. For desethyl-terbuthylazine the $k_{Yn}$ constant is higher than for 2-hydroxy-terbuthylazine. The time required to 50% breakthrough of the column (τ) was significantly longer for 2-hydroxy-terbuthylazine, for desethyl-terbuthylazine corresponds well with experimental data. The correlation coefficient is high only in the case of desethyl-terbuthylazine which may mean this model could be suitable for this metabolite. 

**Figure 9:**
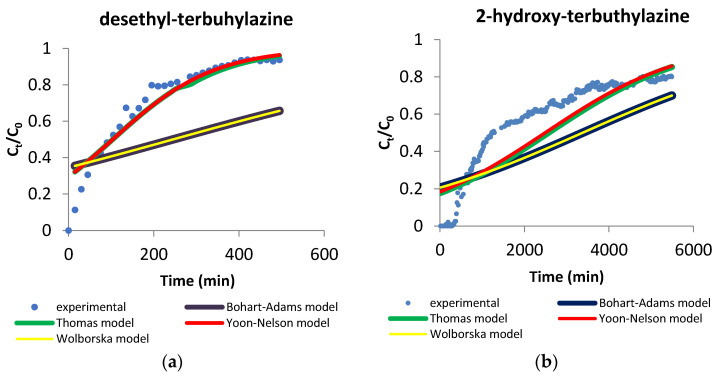
Experimentally obtained breakthrough curves and fixed bed column modeling curves for desethyl-terbuthylazine (**a**) and 2-hydroxy-terbuthylazine (**b**).

The Wolborska model is mainly used to describe breakthrough curves at low concentrations of effluent [59]. Mass transfer may be represented by Equation (12):

(12) $$ln\left(\frac{C_{t}}{C_{0}}\right)=\frac{\beta \cdot C_{0}}{N_{0}}\cdot t-\frac{\beta \cdot Z}{U_{0}}$$

where: $C_{0}$ and $C_{t}$ (mg L^−1^) are the initial and breakthrough concentration, *β* (1 h^−1^) is the kinetic coefficient of the external mass transfer, $N_{0}$ (mg L^−1^) is the exchange capacity, *Z* (mm) is the height of the fixed bed, $U_{0}$ (mm h^−1^) is the superficial fluid velocity. Parameters *β* and $N_{0}$ were determined from linear plot: $ln\left(\frac{C_{t}}{C_{0}}\right)$ versus time (*t*). Determined parameters *β* and $N_{0}$ are higher for 2-hydroxy-terbuthylazine. Correlation coefficients are low for both metabolites, which may mean that kinetics systems were not dominated by external mass transfer [60]. Precise simulation of breakthrough curves may be critical for controlling the effectiveness of adsorbent. It may also be useful for determining the life of adsorption bed and regeneration time or even the adsorption capacities [61]. Figure 9 shows experimentally obtained curves (pH = 7, T = 20 °C) for both metabolites with predicted model curves for every theoretical fixed-bed column model.

The SSE parameters (defined as the sum of the square estimate of errors) were obtained using software Origin Pro [53] and are presented in Table 7. The values are high, which can suggest that analyzed models may not be suitable for such complex systems. It can be seen that for desethyl-terbuthylazine the Thomas and Yoon–Nelson models results may be suitable, whereas in the case of 2-hydroxy-terbuthylazine none of the examined models seem to be correct.

In summary of column adsorption modeling, the correlation coefficients are lowest for the Bohart–Adams and Wolborska models for both metabolites of terbuthylazine, whereas coefficients for the Thomas and Yoon–Nelson models are higher and comparable. Used models are considered simple and may not be effective in describing such complex systems. Accordingly, to obtain more detailed information, other models should be explored.

#### 3.3.3. Desorption Studies 

Desorption process was analyzed based on the mass balance of adsorbed herbicide and then desorbed from the column bed. Investigated herbicide metabolites show good solubility in ethanol, and due to that it was chosen as an eluent in the desorption process. Parameters such as desorption efficiency, amount of eluent and desorption time were determined and are shown in Table 8. Desorption efficiency is higher for desethyl-terbuthylazine and is associated with solubility of metabolites in ethanol. Desorption time was longer for 2-hydroxy-terbuthylazine (315 min) than for desethyl-terbuthylazine (185 min). It was noted that better solubility determines shorter time needed for elution of substances. During this study, it was also observed for both metabolites that after passing small volume of ethanol significant amounts of the herbicides were desorbed, but process was carried out until complete elution from the bed.

**Figure 10:**
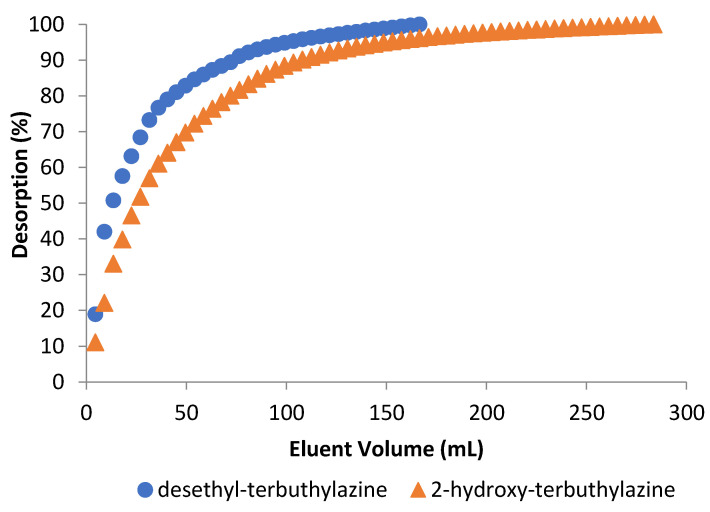
Desorption of terbuthylazine metabolites using ethanol.

Figure 10 shows the desorption efficiency as a function of eluent volume passed through column bed. After passing 13.5 mL of eluent, about 50% of desethyl-terbuthylazine was desorbed, whereas for 2-hydroxy-terbuthylazine 27 mL were needed. 2-Hydroxy-terbuthylazine shows better affinity to used microspheres, which makes this metabolite more difficult to remove from the column bed than desethyl-terbuthylazine. However, we can assume that even the small amount of eluent is enough to regenerate the bed from both herbicides in industrial conditions, which makes the process economically advantageous. 

**Figure 11:**
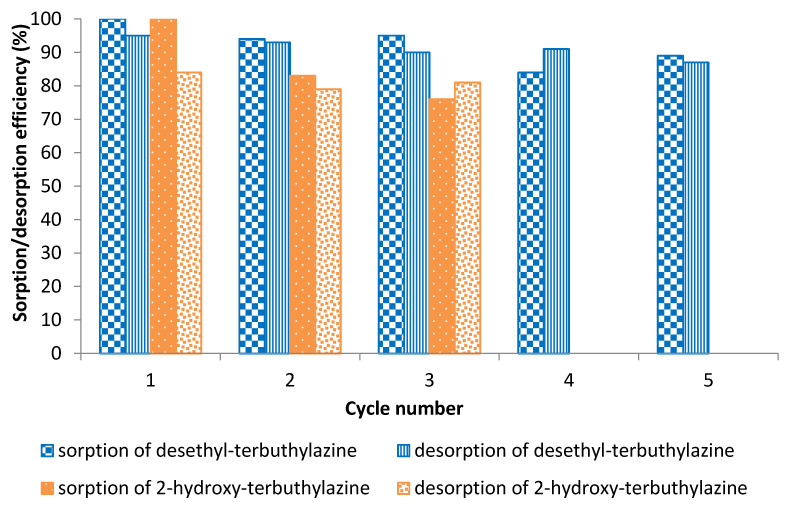
Efficiency of sorption and desorption of studied terbuthylazine metabolites.

Regeneration of polymer bed is important from environmental and also economical point of view. Due to the effective removal of both metabolites of terbuthylazine from the adsorbent it was decided to conduct sorption–desorption cycles. For desethyl-terbuthylazine five cycles were performed, whereas only three for 2-hydroxy-terbuthylazine due to the very long time needed for that process. Ethanol was used as eluent and after passing this solvent, the polymer bed was washed using distilled water. Analyzing the obtained results from the sorption–desorption cycles, it was noted that removal of both herbicides was still very effective. The results are shown in Figure 11. 

In the case of desethyl-terbuthylazine, adsorption efficiencies in first three cycles are very high (up to 100% removal was achieved) and comparable. In the 4th and 5th cycles the adsorption efficiency is slightly reduced to about 90%. The efficiency of desorption is about 90% and is comparable in five cycles conducted. From sorption-desorption cycles of 2-hydroxy-terbuthylazine it can be noted that sorption efficiency is reduced by almost 20% in the second cycle. The third cycle showed adsorption efficiency reduced by 8% compared to the second cycle. Desorption efficiency is comparable in every cycle and is about 80%. Those results confirm the possibility of using poly(divinylbenzene) modified with maleic anhydride many times not only in the detection of herbicides, but also in water treatment. 

## 4. Conclusions

The proposed polymeric microspheres are suitable for the sorption of selected terbuthylazine metabolites. We observed that the differences in the physicochemical properties of the compounds caused five times greater sorption of 2-hydroxy-terbuthylazine than desethyl-terbuthylazine. The kinetic data for both metabolites fit well to a pseudo-second order kinetic model, which suggests that the adsorption rate is dependent on the availability of adsorption sites more than on herbicide concentration. The equilibrium data in the batch study follow the Freundlich isotherm for 2-hydroxy-terbuthylazine, and for desethyl-terbuthylazine the Temkin and Dubinin–Radushkevich models are suitable. The Dubinin–Radushkevich isotherm model suggests that processes of adsorption are physical in both systems, while the Temkin model confirms their exothermic character. The adsorption of desethyl-terbuthylazine does not depend on the pH. For 2-hydroxy-terbuthylazine, which is even much better adsorbed than its parent compound, the intensity of adsorption obtained under acidic conditions was considerably smaller (comparable with sorption capacity for desethyl derivative); however, such results can still be considered satisfactory. The results of this study also show that hydrogen bonds have a significant impact on sorption efficiency. These specific interactions are also responsible for the selectivity of sorption. For this reason, the proposed microspheres should work effectively in environmental conditions. The adsorption capacities obtained under dynamic conditions were comparable with batch results. In column adsorption modeling using the Bohart–Adams, Wolborska, Thomas and Yoon–Nelson models, a good fit for the Yoon–Nelson model was observed for desethyl-terbuthylazine. For 2-hydroxy-terbuthylazine other models should be explored. Column studies have shown that adsorbed metabolites of terbuthylazine can be easily desorbed by ethanol, and due to that fact, the modified poly(divinylbenzene) can be used several times without a major change in adsorption capacity. 

The satisfactory sorption efficiency both in static and dynamic conditions suggests that poly(divinylbenzene) modified with maleic anhydride may be successfully used in terbuthylazine metabolites detection and monitoring using solid-phase extraction, specifically as a filling of SPE columns. Probably, it could also be used as packing material for a chromatographic column dedicated to triazine herbicide measurements. Moreover, it can be also used successfully in removing these persistent and mobile compounds from aquatic environments.

## Figures and Tables

**Table 1:** The physicochemical properties of analyzed terbuthylazine metabolites.

Herbicide	Structure	Molecular Weight	Solubility in Water 20 °C (mg L^−1^)	pK_a_ * [41]	logK_ow_ *
desethyl-terbuthylazine	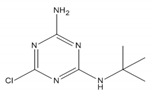	201.66	327.1 [42]	~2	1.94 [43]
2-hydroxy-terbuthylazine	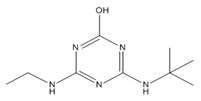	211.26	7.19 [44]	~5	2.54 [45]

* pK_a_, acid dissociation constant; K_ow_, octanol/water partition coefficient.

**Table 2:** Parameters of pseudo-first order and pseudo-second order adsorption kinetic model.

Terbuthylazine Metabolite	Pseudo-First Kinetic Model			Pseudo-Second Kinetic Model		
	*R* ^2^	*q_eq_* (mg g^−1^)	*k*_1_ (min^−1^)	*R* ^2^	*q_eq_* (mg g^−1^)	*k*_2_ (g mg^−1^ min^−1^)
desethyl-terbuthylazine	0.965	13.9	$1.8\times 10^{-3}$	0.975	16.4	$1.2\times 10^{-4}$
2-hydroxy-terbuthylazine	0.910	42.4	$1.2\times 10^{-3}$	0.991	115	$1.4\times 10^{-4}$

**Table 3:** Sorption capacity (*q_eq_*), retention rate (*R*) and distribution coefficient (*K*) for adsorbed terbuthylazine metabolites.

Terbuthylazine Metabolite	*q_eq_* (mg g^−1^)	*R* (%)	*K* (-)
desethyl-terbuthylazine	21	80	2800
2-hydroxy-terbuthylazine	113	88	24,400

**Table 4:** Parameters of Langmuir, Freundlich, Temkin and Dubinin–Radushkevich isotherm models.

Isotherm Model	Parameter	Metabolite	
		desethyl-terbuthylazine	2-hydroxy-terbuthylazine
Langmuir	*q_m_* (mg g^−1^)	263	476
	*K_α_* (dm^3^ mg^−1^)	0.01	0.05
	*R* ^2^	0.969	0.916
	*SSE* *	$2\times 10^{-4}$	$4\times 10^{-5}$
Freundlich	1/*n*	1.37	1.01
	*K_F_* ((mg g^−1^)(L mg^−1^)^1/*n*^)	1.98	20.81
	*R* ^2^	0.949	0.913
	*SSE* *	0.058	0.026
Temkin	*A_T_* (L g^−1^)	0.61	1.05
	*b_T_* (J mol^−1^)	201	40
	*R* ^2^	0.989	0.834
	*SSE* *	4.03	1051.26
Dubinin-Radushkevich	*q_s_* (mg g^−1^)	21.6	94.9
	*K_ad_* (mol^2^ J^−2^)	$2\times 10^{-6}$	$6\times 10^{-7}$
	*E* (kJ mol^−1^)	0.5	0.9
	*R* ^2^	0.970	0.731
	*SSE* *	0.12	0.23

* *SSE*, Sum of Square Estimate of Errors.

**Table 5:** Mathematical equations of the breakthrough curve for investigated terbuthylazine metabolites.

Terbuthylazine Metabolite	Mathematical Equation of the Breakthrough Curve	*R* ^2^
desethyl-terbuthylazine	y = 203.32x^5^ − 790.86x^4^ + 774.9x^3^ − 345.84x^2^ + 82.888x + 0.1131	0.996
2-hydroxy-terbuthylazine	y = 0.0056x^5^ − 0.1269x^4^ + 1.0373x^3^ − 4.074x^2^ + 8.6167x − 1.18137	0.987

**Table 6:** Characteristic parameters of dynamic sorption obtained in the tested systems.

Terbuthylazine Metabolite	Dynamic Adsorption Capacity (mg g^−1^)		Bed OperationTime until Breakthrough *t_b_* (h)	Bed Operation Time until Exhausted *t_e_* (h)	Height of Adsorption Front *H*_0_ (cm)	Mass Exchange Moving Rate *u* (cm min^−1^)
	*P_t_* (Total)	*P_u_* (Useful)				
desethyl-terbuthylazine	10	1	0.25	5.75	3.13	$9.5\times 10^{-3}$
2-hydroxy-terbuthylazine	113	39	8.50	75.75	2.80	$6.1\times 10^{-4}$

**Table 7:** Parameters of Adams–Bohart, Thomas, Yoon–Nelson and Wolborska models.

**Model**	Parameter	Metabolite	
		desethyl-terbuthylazine	2-hydroxy-terbuthylazine
Adams–Bohart	$k_{AB}$ (L mg^−1^ min^−1^)	$2.60\times 10^{-4}$	$4.00\times 10^{-5}$
	$N_{0}$ (mg L^−1^)	4254.5	45,294.4
	*R* ^2^	0.635	0.385
	*SSE* *	2.63	146.21
Thomas	$k_{Th}$ (mL mg^−1^ min^−1^)	$8.40\times 10^{-4}$	$6.00\times 10^{-5}$
	$q_{0}$ (mg g^−1^)	$1.13\times 10^{4}$	$2.73\times 10^{5}$
	*R* ^2^	0.915	0.606
	*SSE* *	4.39	185.40
Yoon–Nelson	$k_{Yn}$ (min^−1^)	$8.40\times 10^{-3}$	$6.00\times 10^{-4}$
	τ (min)	103.6	2489.8
	*R* ^2^	0.915	0.606
	*SSE* *	4.93	185.40
Wolborska	*Β* (1 h^−1^)	0.0184	0.0332
	$N_{0}$ (mg L^−1^)	1.18	15.24
	*R* ^2^	0.635	0.385
	*SSE* *	2.63	146.45

* *SSE*, Sum of the Square Estimate of Errors.

**Table 8:** Characteristics of terbuthylazine metabolites desorption process.

Terbuthylazine Metabolite	Desorption Efficiency (%)	Amount of Eluent (mL)	Desorption Time (min)
desethyl-terbuthylazine	95	166	185
2-hydroxy-terbuthylazine	84	284	315

## Data Availability

The data presented in this study are available on request from the corresponding author.
